# Diversity and Relationships among Neglected Apricot (*Prunus armeniaca* L.) Landraces Using Morphological Traits and SSR Markers: Implications for Agro-Biodiversity Conservation

**DOI:** 10.3390/plants10071341

**Published:** 2021-06-30

**Authors:** Giandomenico Corrado, Marcello Forlani, Rosa Rao, Boris Basile

**Affiliations:** 1 Department of Agricultural Sciences, University of Naples Federico II, 80055 Portici, NA, Italy; marcello.forlani@unina.it (M.F.); rosa.rao@unina.it (R.R.); boris.basile@unina.it (B.B.); 2 Consorzio Interuniversitario Biotecnologie (CIB), University of Naples Federico II Unit, 80055 Portici, NA, Italy

**Keywords:** stone fruit, local varieties, germplasm, pomological traits, DNA fingerprinting, microsatellites

## Abstract

Apricot (*Prunus armeniaca* L.) is an economically important tree species globally cultivated in temperate areas. Italy has an ample number of traditional varieties, but numerous landraces are abandoned and at risk of extinction because of increasing urbanization, agricultural intensification, and varietal renewal. In this work, we investigated the morphological and genetic diversity present in an ex-situ collection of 28 neglected varieties belonging to the so-called “Vesuvian apricot”. Our aim was to understand the level of diversity and the possible link between the promotion of specific fruit types (e.g., by public policies) and the intraspecific variation in apricot. The combination of five continuous and seven categorical traits allowed us to phenotypically distinguish the varieties; while fruit quality-related attributes displayed high variation, both apricot size and skin colour were more uniform. The twelve fluorescent-based Simple Sequence Repeats (SSRs) markers identified cultivar-specific molecular profiles and revealed a high molecular diversity, which poorly correlated with that described by the morphological analysis. Our results highlighted the complementary information provided by the two sets of descriptors and that DNA markers are necessary to separate morphologically related apricot landraces. The observed morphological and genetic differences suggest a loss of diversity influenced by maintenance breeding of specific pomological traits (e.g., skin colour and size). Finally, our study provided evidence to recommend complementary strategies to avoid the loss of diversity in apricot. Actions should pivot on both the promotion of easily identified premium products and more inclusive biodiversity-centred on-farm strategies.

## 1. Introduction

Apricot (*Prunus armeniaca* L.) is a stone-fruit tree globally appreciated for the rich flavour, fragrant aroma, and versatility of use of the drupes. The juicy, firm, and nutritious fruits can be consumed fresh, dried, in syrup, or made into jams [1]. Apricot was domesticated in Central Asia and was spread across Western Europe by the Romans [2,3]. It is no coincidence that the word ‘apricot’ allegedly derives from the Latin *arbor praecox* (the tree with an early production) [2]. The temperate climate and the varied orographic conditions favoured the diffusion of apricot in Italy and this species has experienced a considerable diversification, whose heritage is still visible. According to the International Plant Genetic Resources Institute, Italy has the largest collection of apricot varieties [4]. 

Italy is one of the top world producers of apricots [5] and the Campania region (Southern Italy) has the largest cultivated area, providing around one-third of the entire national production [6]. In this region, the empirical selection of the growers has created a wealth of landraces that are distributed mainly in the Naples Province [7], mostly because apricot has been one of the typical cultivations of the South-facing, rain-fed Vesuvian slopes since Roman times [8]. This rich germplasm is often referred to as Vesuvian apricots [9,10]. Nonetheless, the Neapolitan word from apricot (*cresommela*) originates from Greek [χρυσό μήλο; golden fleshy (tree) fruit], suggesting that the introduction of this tree in this area could date back to the settlers of the Magna Graecia era [3].

In the last decade, apricot has provided satisfactory economic results for the entire supply chain (including sweet manufacturing) when compared to other stone fruits. The apricot sector was sustained by a strong varietal innovation driven by consumer demand for fruits with strongly coloured skin [11]. Therefore, the preference of apricot growers has experienced a transition from varieties that are also suitable for processing to the ones employed only for the fresh market. Specifically, vast attention has been given to new cultivars, often introduced from foreign countries, that have fruits with an intense red skin over colour (red blush), and to those that allow the extension of the harvesting period [12]. On the other hand, apricot is considered a species with reduced environmental adaptability, and the introduction of exotic germplasm may also result in fluctuating or limited yield. This is associated with differences in fertilization, chilling requirements, late-frost resistance, cold-hardiness, and in certain instances, the need for specific cultural techniques [13]. Especially in Southern Italy, there are problems in introducing contemporary self-incompatible, freestone varieties in areas where chilling requirements cannot be always fully satisfied, an issue of rising importance in the face of climate change [14,15]. For all these reasons, traditional cultivars in the Campania region still provide interesting economic results in local markets, remaining a popular option in small farms (<5 ha) and, more recently, in agritourism and farm stay enterprises [16]. Although yield and resistance to mechanical injury may not always be ideal, the locally adapted landraces are appreciated for their superior flavour and aroma [17], leading to the request of the EU Protected Geographical Indication (PGI) “Albicocca Vesuviana” (Vesuvian apricot) label. Moreover, apricots cannot be stored for a long period, leaving room for Short Food Supply Chains as alternative promoters of agricultural, social, and economic sustainability [18]. 

The potential of traditional or neglected varieties to diversify the apricot sector, to support local producers, and to promote traditional gastronomic products that use dried and candied fruits has not gained momentum because of the lack of information on available plant material [17,19,20]. Knowledge of the characteristics and variability of the apricot landraces is central for the selection and promotion of premium products [21,22,23]. It is therefore necessary to fill the gap between the available diversity and the folk names recognised by local consumers [24]. In addition, the evaluation of the apricot landraces is also a measure to indirectly support and acknowledge farmers in return for their precious role in promoting agro-biodiversity and, specifically for the Vesuvian germplasm, in sustaining rural areas in the most densely populated volcanic region in the world.

The objective of this study was to characterize and evaluate the diversity of 28 traditional apricots cultivated in the Campania region. Specifically, our work aimed at addressing the potential impact of a pattern of specialization in the local apricot market over landraces diversity. We used 12 morphological traits and 12 fluorescent based-SSR molecular markers to offer a more comprehensive view of the variation present in the germplasm. Morphological descriptors allow a technically undemanding evaluation of the diversity and represent an easily adaptable classification approach, while DNA fingerprinting is an indispensable tool to assess genetic diversity, discriminate varieties, identify possible synonyms and homonyms, and genetically trace plant varieties in food chains [25,26,27].

## 2. Results

### 2.1. Morphological Analysis

To assess the morphological diversity in the germplasm collection, we scored five multistate categorical traits and seven quantitative traits of the fruit (Supplementary Table S1). All morphological traits were polymorphic, presenting two or more different forms (Table 1 and Table 2). The most variable qualitative pomological trait was the colour of the flesh, considering the scored phenotypes, their distribution, and the Simpson Index of Diversity (SDI). Little variation was present for the ground colour of the skin. We scored only two and similar ground colour of the seven possible categories listed in the UPOV guidelines. A single predominant phenotype was not evident, and the SDI was relatively high (0.48), indicating a good distribution of the abundance of this trait. Very little variability was present for the adherence of the stone to the fruit and the kernel bitterness (SDI: 0.07). Nine varieties presented a unique combination of qualitative traits; nonetheless, the most common phenotype (a strongly vigorous tree producing fruits with a yellowish ground skin colour and a medium-orange pulp, and with a bitter, free stone) was present only in four varieties.

The range of variation of the quantitative traits is presented in Table 2. The average coefficient of variation (CV) was 22.2, indicating the presence of considerable differences in the pomological traits. 

**Figure 1:**
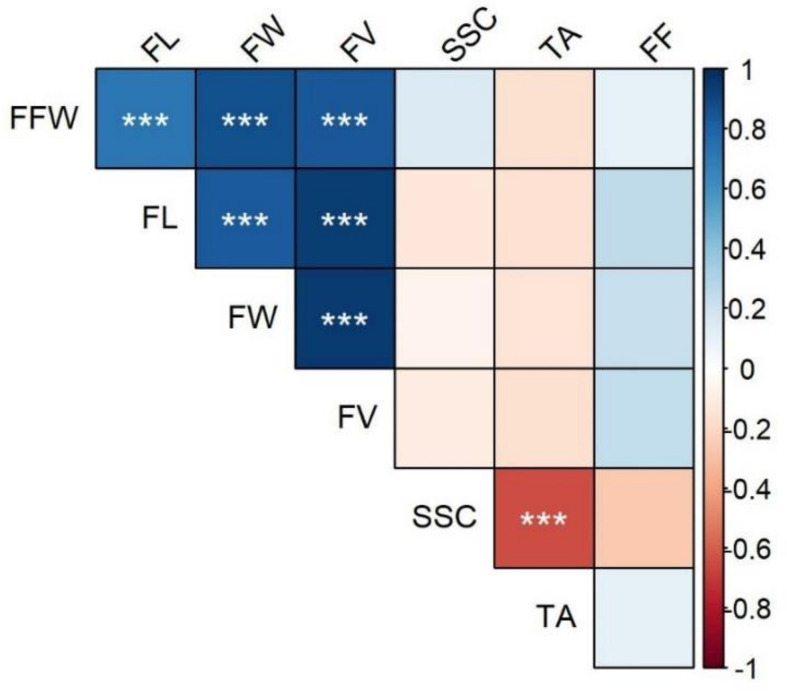
Correlogram (Pearson) of the quantitative variables of the fruits. Pairwise correlations between variables (see Table 2. for the code) are colour-mapped according to the colour scale of the bar on the right-hand side. Asterisks indicate statistically significant correlations (***: *p* < 0.001).

Traits displayed substantial differences in their range of variation. For instance, fruits varied slightly in length and width. The fruit volume was the most variable morphological feature, but its variation (~27%) was proportionate to the extent of the linear measurements (~9%) in three dimensions. The greatest differences were evident for fruit quality, with the titratable acidity having the highest CV, followed by the flesh firmness and the soluble solid content. The presence of a rather uniform fruit shape is indirectly indicated by the strong positive correlation between width, length, and volume of the fruit, while quality-related pomological traits displayed non-significant correlations, with the notable exception of the negative value for SSC and TA (Figure 1).

**Figure 2:**
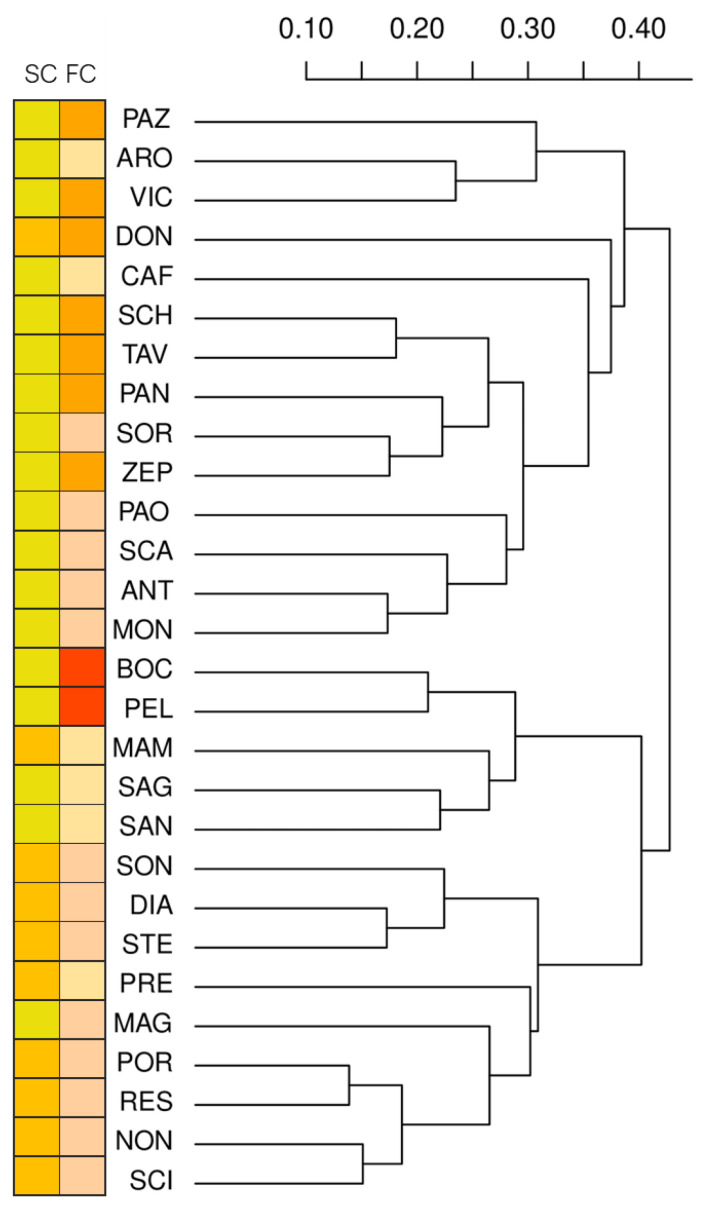
Hierarchical cluster analysis of the 28 apricot landraces based on the 12 scored traits. Distances were computed with the Gower’s coefficient. The coloured squares on the left indicate the ground skin colour (SC) and flesh colour (FC) of the fruits (data are reported in Supplementary Table S1).

To visualize the relationships among the apricot landraces under investigation, we performed a multivariate cluster analysis using both qualitative and quantitative traits. At a high hierarchical node (k = 2), the top cluster (PAZ-MON) was associated with a yellowish skin colour (13 varieties out of 14), while the bottom (BOC-SCI) mainly with the light orange one (9 out of 14) (Figure 2).

This explorative analysis also indicated that cutting the tree at k = 11 highlights groups of landraces with similar skin and fruit flesh colour. For instance, the cluster SCH-ZEP (five varieties) was characterised mostly by yellowish skin colour and a medium orange flesh, while the cluster PAO-MON (four varieties) differed because of light orange flesh. Other phenotypically distinct clusters were the BOC-PEL (two varieties; yellowish skin and dark orange flesh), the SON-STE (three varieties; light orange skin and flesh colour) and the MAG-SCI cluster (five varieties; mostly with light orange skin and with light orange pulp). Overall, the morphological analysis indicated that the germplasm under investigation presented some distinctive features, considering the trait variation described in apricot. Albeit the varieties could be distinguished based on both quantitative and qualitative traits, the germplasm under investigation was largely characterized by medium size, obovate fruits with not strongly coloured fruits and flesh. Moreover, the association of a fruit phenotype with agglomerative clusters suggests a possible similar origin for at least some varieties.

### 2.2. Analysis of Genetic Diversity by SSR Markers

The genetic diversity was assessed using 12 SSRs selected from the literature as specific to apricot. The loci were all polymorphic, and the main genetic parameters are presented in Table 3. We detected in total 76 alleles and their length (from 79 to 300 bp), was consistent with the literature [19,28,29]. Differences among loci were in the number of alleles, which ranged from 2 (AMPA111) to 10 (UDAp-446). Considering the effective number of alleles (i.e., the number of alleles weighted for their frequencies), the most diverse locus was AMPA112, followed by UDAp-410 and UDAp-446. Not surprisingly for an agamically propagated species, the observed heterozygosity (Ho) was high (mean value: 63%) but large variations were present among loci. The number of alleles was positively associated with the Ho, but the correlation was not significant (r_s_ = 0.32, *p* (two-tailed) = 0.16, Spearman’s rho). Specifically, heterozygotes were not present for one locus (AMPA111), which was fixed in our population. The transcript in the *P. armeniaca* genome (Seq_id: tig00008589_30087 in the assembly 1.0) closest to AMPA111 (distant approximately 400 bp) putatively codes for a protein that has the highest similarity (blastx e-value: 7 × 10^−37^; similarity: 47%) with a zeaxanthin epoxidase from *P. mume* (XP_008224462.2), which is involved in carotenoid accumulation [30]. On the other hand, UDAp-419 also displayed a substantial positive Fixation Index (0.63) yet this locus had eight different alleles. As expected, the Polymorphic Information Content of the loci significantly correlated with the number of alleles (r_s_ = 0.96, *p* (two-tailed) < 0.001, Spearman’s rho). Three loci (UDAp-446, AMPA112, and UDAp-410) were almost equally highly informative, considering the Information index, the number of alleles, and PIC. Finally, the rate of proportional abundance homogeneity of individual alleles in the population was high for all loci, as indicated by the Evenness values, which were negatively correlated with the number of alleles (r_s_ = -0.63, *p* (two-tailed) < 0.05, Spearman’s rho).

**Figure 3:**
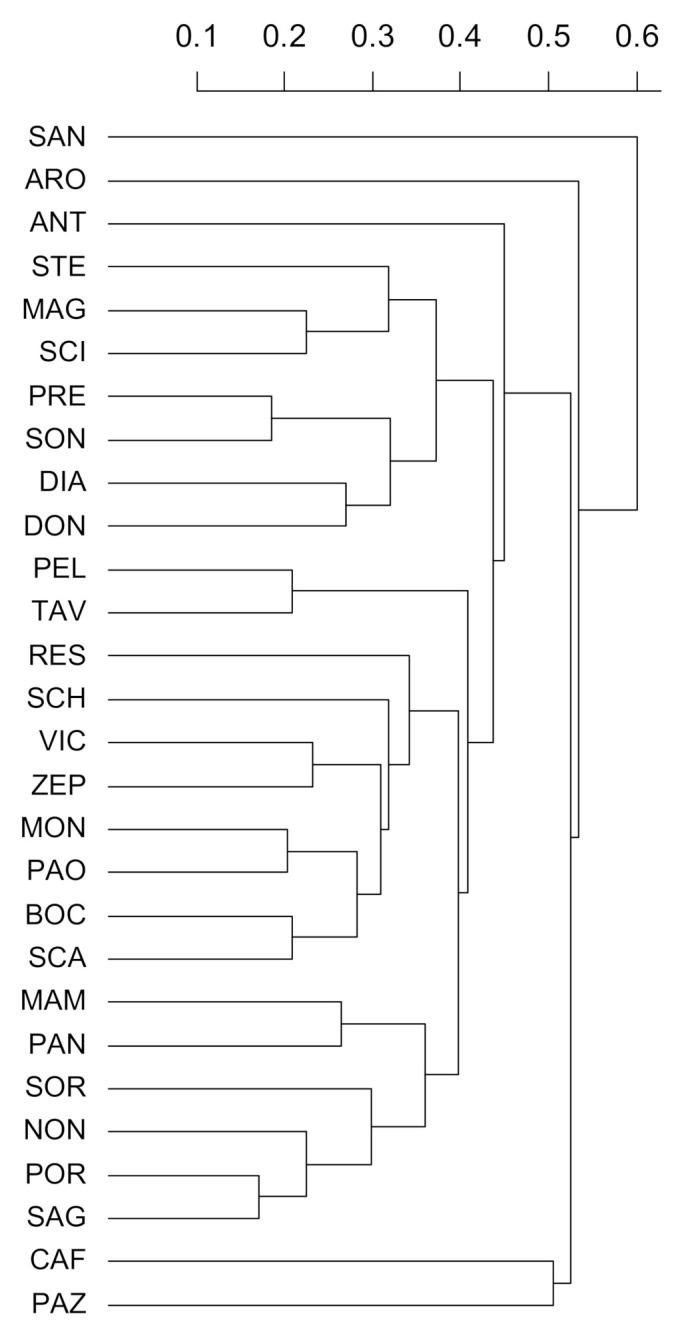
Cluster analysis (UPGMA algorithm based on Bruvo’s distances from SSR data) of the 28 apricot landraces. The scale bar for the genetic distance is presented on the top.

To evaluate the genetic relationship between varieties, we built a UPGMA dendrogram (Figure 3). Genetic distances are reported in Supplementary Table S2. All the varieties could be discriminated, and the average (± standard deviation) genetic distance was 0.44 ± 0.11. Moreover, a clear tendency in grouping phenotypically similar varieties was not evident. For instance, varieties with similar fruit or flesh colour did not clearly agglomerate according to the genetic analysis. Overall, the dendrogram based on molecular data did not identify groups shown by the morphological analysis.

**Figure 4:**
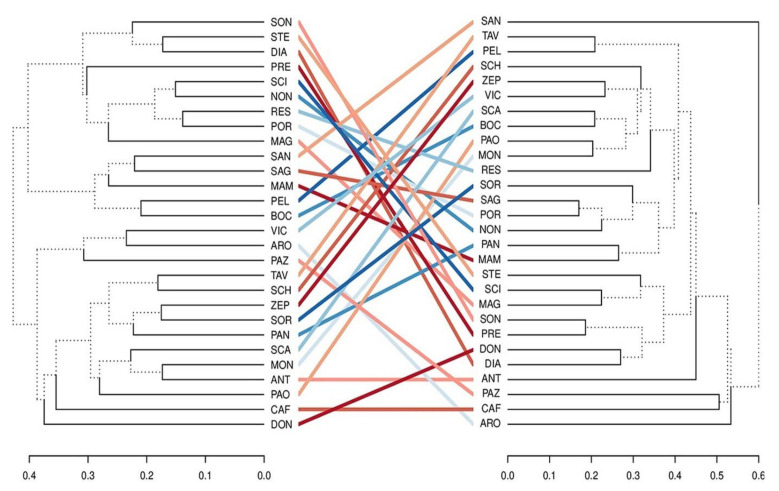
A comparison of the hierarchical clustering of the apricot varieties using morphological (left tree) or SSR marker (right tree) data. Clustering was performed with the UPGMA algorithm for both dendrograms. To ease the comparison, coloured lines connect identical names. The different line types in the dendrograms highlight distinct edges in a tree (compared to the other one).

To better visualize the differences between morphological and molecular data in describing diversity, we compared dendrograms. The correlation between the distances obtained with the two datasets (r = 0.18; *p* > 0.05; Mantel test with 9999 permutations) and the cophenetic correlation between the dendrograms (r = 0.19) were not significant. The tanglegram indicated that the resemblance depicted with the morphological traits did not largely correlate with that obtained with SSR markers (Figure 4).

## 3. Discussion

The market promotion of the traditional plant varieties necessitates a two-way transfer of knowledge between farmer–custodians and extension specialists [31], sharing the common goal of enhancing landraces’ symbolic and socio-cultural value and their profitability. To raise stakeholder engagement, it is needed a thorough description and discrimination of the germplasm’s features to go beyond, and hopefully reduce, the still essential strategy of providing economic incentives to maintain crop landraces on-farm [32,33]. Moreover, the recognition of the existing diversity is a prerequisite to define policies for agricultural biodiversity [34,35] and to increase consumer loyalty, at least in local markets and for selected traditional products [36].

Our study revealed the phenotypic and genetic diversity in the virtually neglected apricot germplasm under investigation. Several phenotypes were scored, but there was limited variability in the ground skin colour of the fruits considering the UPOV categories and the literature [37,38,39]. Different colours in local germplasm (e.g., green-yellowish colour for ‘Persicara’; dark orange for ‘Cerasona’ and ‘Parrocchiana’) have been reported in non-scientific and grey literature, suggesting a possible loss of diversity. In our germplasm, higher variation was evident for the flesh colour and tree habit, while the bitterness of the kernel and the adherence of the stone displayed a similar frequency (e.g., a largely predominant phenotype) than in other apricot collections [38,40]. All this can be explained considering that the observable pomological characteristics have clear commercial importance, and therefore, these traits are more likely to be objects of maintenance breeding [41]. Traditional local varieties of the Campania region, and especially those considered typical of Mount Vesuvius, are associated with a light orange-yellowish colour, medium weight, and an elliptic/obovate shape [42]. The recognition of the typical phenotype of the Vesuvian apricot, and related market demand, could have been important drivers for maintenance breeding in the face of biodiversity erosion. The apricot cultivation in the Campania region largely changed in the second half of the XIX century, where in a rentier agricultural economy, there was a shift towards the production of large quantities of standardized apricots to satisfy the demand of a rising, low-income, urban population of Naples, at that time, the third largest European city [43,44]. More recently, the cultivation of apricot has been limited by the anthropization of a vast portion of the peri-urban hilly areas of the Naples Province [45].

Hierarchical analysis indicated that different clusters could be associated with some qualitative pomological traits, although the quantitative traits under investigation were higher in number and with good variability. For instance, the variation of TSC and TA was higher compared to traditional apricot landraces and similar to that reported in contemporary cultivars [39,46], implying that the traditional germplasm can be a potential source of variation of quality-related fruit traits [12]. This is relevant because, different from other works [37,46,47], we analysed plants growing in the same environment and with the same cultural practices [39], allowing us to untangle the genetic and environmental contribution to quantitative trait variation.

Another difference from previously published works is that our cluster analysis jointly considered qualitative and quantitative traits [37,47]. Moreover, in our work, quantitative traits were first standardized (but not weighted), not only to share a common scale but also for their different biological meaning and unit of measurement [48]. The morphologically similar subclusters prompted us to test a possible common ancestry by using DNA molecular markers. It is known that traditional apricot varieties in small farms are also selected from seedlings, leaving open the possibility of a common origin of at least some of the clusters that show a similar pomological appearance. The molecular analysis indicated a high level of molecular diversity, comparable to that of other reports on larger numbers of landraces [49], or higher than in similarly sized collections [50]. For instance, the SSR loci showed an average level of heterozygosity that was in line with previous work, but locus-specific differences in some genetic parameters were also evident [51]. One locus (AMPA111) was fixed and another (UDAp-419) had a fixation index higher than 0.5. While the former had two evenly distributed alleles, the latter was characterized by a high number of alleles. It is difficult to speculate on the reasons for these locus-specific differences and, specifically, on the AMPA111 fixation. The agamic propagation preserves both adaptive and neutral genetic diversity, thus affecting both the distribution of alleles at loci under selection and that of neutral loci. The limited variability in fruit skin colour makes it tempting to speculate a possible selection-driven fixation of a locus tightly associated with a carotenogenic gene [30] however, further genetic and biochemical studies will have to clarify the adaptive significance of these variations, along with the possible presence of a founder effect, or of genetic drift. For all SSR loci, the number of alleles was within, if not higher than the range reported in the literature [28,29,52,53]. Distinct DNA profiles could be identified for each variety, without possible cases of synonymy and/or duplicated accessions that can be present in traditional germplasm [52,54]. This may be explained considering that the analysed germplasm belongs to an ex-situ collection, whose material was previously classified based on farmers’ description. For all these reasons, the molecular analysis does not favour the possibility that the similar-looking varieties have a recent common origin, deriving for instance, from the selection of seedlings of open-pollinated plants from neighbouring farms [51]. The data suggested that the frequency of plants with a similar pomological appearance is due to the selection of aesthetically important indicators of marketability.

Finally, the comparison between morphological and molecular diversity indicated that morphological descriptors in apricot provide different classifications from the molecular ones, consistent with studies in other tree crops [55,56]. Our results imply that in apricot landraces, an estimation of the variability exclusively based on morphological traits can misrepresent the level of diversity, and therefore, DNA markers can be very useful for building core collections in apricot [57]. Moreover, the assignment of trees to a variety, when based exclusively on morphological similarity, would require scoring a high number of both qualitative and quantitative traits, especially to avoid possible spurious homonymies.

## 4. Materials and Methods

### 4.1. Plant Material

The work was carried out on 28 *Prunus armenicaca* L. landraces present in the germplasm collection of the Azienda Agricola Sperimentale Regionale Improsta, Centro per la Ricerca Applicata in Agricoltura (C.R.A.A.). The varieties (abbreviation) were: ‘Antonaniello’ (ANT), ‘Aronzo’ (ARO), ‘Boccuccia liscia II’ (BOC), ‘Cafona’ (CAF), ‘Diavola’ (DIA), ‘Don Aniello’ (DON), ‘Magnalona’ (MAG), ‘Mammana’ (MAM), ‘Montedoro’ (MON), ‘Nonno’ (NON), ‘Panzona’ (PAN), ‘Paolona’ (PAO), ‘Pazza’ (PAZ), ‘Pelese Correale’ (PEL), ‘Portuallara’ (POR), ‘Presidente’ (PRE), ‘Resina’ (RES), ‘San Giorgio’ (SAG), ‘Sant’Antonio’ (SAN), ‘Scassulillo’ (SCA), ‘Schiavona’ (SCH), ‘Scialo’ (SCI), ‘Sonacampana’ (SON), ‘Sorrentino’ (SOR), ‘Stella’ (STE), ‘Taviello’ (TAV), ‘Vicienzo (syn: Vicienzo ‘e maria)’ (VIC), ‘Zeppa (syn: Zeppa ‘e sisco)’ (ZEP). These locally cultivated varieties were selected because they are considered at risk of extinction since they were not included in the proposal for the EU Protected Geographical Indication (PGI) “Albicocca Vesuviana” (Vesuvian apricot) (Gazzetta Ufficiale della Repubblica Italiana, Serie Generale n.66, 19-03-2002). These crop varieties can be considered “neglected” according to the literature [58], because in the past they were of greater importance in traditional agriculture, in the diet of local communities, and in local food processing activities [59]. Subsequently, they have been marginalized because of the introduction of contemporary varieties and gradual change in consumer demand, as well as for economic, societal, and cultural factors that contribute to the disappearance of social groups that cultivated this material [58].

### 4.2. Analysis of Morphological Data

Tree vigour was visually evaluated in the field and assigned to one of the following categories: very weak, weak, medium, strong, very strong, according to the document TG/70/4 Rev. (proj.2) of the International Union for the Protection of new Varieties of Plants (UPOV, Geneva, Switzerland). Not available (NA) was assigned in the cases of off-types and lack of uniformity. A sample of 30 fruits per variety (six fruits from five plants) was collected and used to assess the following morphological traits: fresh weight, height, ventral width, lateral width, volume, skin ground colour, flesh colour, flesh firmness, soluble solids content, titratable acidity, kernel bitterness, and stone adherence to the flesh. Fruit fresh weight was measured with a digital scale, whereas fruit height, ventral width, and lateral width were measured with a digital caliper. For each fruit, mean fruit width was calculated averaging ventral and lateral widths. Single fruit volume was estimated assuming the apricot fruit had an ellipsoidal shape and considering the height and the ventral and lateral widths as axes. Ground skin and flesh colour were evaluated visually, and the fruits were assigned to one of the following possible categories: (a) ground colour of the skin: not visible, white, yellowish, yellow green, light orange, medium orange, and dark orange; (b) flesh colour: whitish green, white cream, light orange, medium orange, and dark orange. Flesh firmness (N) and the soluble solids content in fruit (°Brix) were measured with a digital fruit firmness tester (model #53205, TR, Forlì, Italy) fitted with an 8-mm diameter plunger and a digital refractometer (HI96811, Hanna Instruments, TX, USA), respectively. Titratable acidity was measured adding a 0.1 N NaOH solution to filtered fruit juice until reaching a pH of 8.2. During titration, pH was measured continuously with a laboratory pH-meter (GLP 21; Crison, Alella, Barcelona, Spain). Titratable acidity was expressed in grams per liter of malic acid (g/L). Before juice extraction, fruits were split along the suture to visually evaluate the adherence of the stone to the flesh, classified as present or absent–very weak. Bitterness of ground kernels was also dichotomously classified as present or absent–very weak. The Simpson Index of Diversity (SDI) of each qualitative trait was calculated as 1-D, where D is the sum of n_i_(n_i_ − 1)/N(N − 1); n_i_ is the number of varieties having the ith-phenotype, and N is the total number of varieties. Hierarchical cluster analysis, using the Unweighted Pair Group Method with Arithmetic Mean (UPGMA) algorithm based on Gower’s distance of the unweighted and scaled variables, was carried out as already reported [60]. We did not assign a weight to each trait (or each category of traits), and quantitative data were normalized (Z-score) because variables are on different scales.

### 4.3. DNA Isolation and SSR Analysis

Genomic DNA was isolated from young leaves stored at −80 °C. Powdered leaves (approx. 500 mg) were mixed with 15 mL of warm (65 °C) extraction buffer [61] and after the first precipitation of nucleic acids [61], the pellet was solubilised in 400 µL Buffer AP1 (Qiagen). The DNA was then purified according to the instructions of the DNeasy Plant Mini Kit (Qiagen). Polymerase Chain Reaction (PCR) amplifications were carried out using 12 apricot SSRs primer pairs selected from the literature and reported in the Supplementary Table S1 [19,28,29]. We genotyped two plants per variety, which provided an identical profile for each landrace. PCR reactions (25 µL final volume containing 100 ng of genomic DNA) were performed as previously described [62] using the annealing temperatures listed in Supplementary Table S3. Amplicons were resolved in an agarose gel-electrophoresis to verify the presence of and to quantify the amplified fragments. Allelic discrimination was performed by fluorescence-based capillary electrophoresis using an ABI PRISM 3130 Avant (Applied Biosystems, Milan, Italy) and the POP4 polymer (Applied Biosystems). Allele sizes were calculated with GeneScan 4.0 (Applied Biosystems) software using the local Southern algorithm, as previously described [62]. The raw size was rounded to an integer and scaled considering the number of bases of the repeated core motif (Supplementary Table S3). Alleles were binned, minimizing the mean offset of the allelic size for each SSR within the resolution of the instrument (±1 bp).

### 4.4. Molecular Data Analysis

We calculated, for each SSR locus, the number of different alleles (N), the Shannon’s information index (I), the observed heterozygosity (Ho), and the Polymorphic Information Content (PIC), equivalent to the expected Heterozygosity) as previously reported [56]. The effective number of alleles (ENA), Evenness (Ev), and pairwise genetic distances between varieties using Bruvo’s coefficient were computed with the poppr R-library [63]. Hierarchical clustering (UPGMA algorithm) was carried out as previously described [60]. To test the correlation between the morphological and molecular data, the two parallel matrices were compared by a Mantel test (9999 permutations) [60].

## 5. Conclusions

Our work indicated that the combination of molecular and morphological data for the classification of traditional apricot varieties is probably necessary to separate morphologically related accessions, an important issue particularly for local germplasms subject to a possible genetic erosion because of the selection for specific fruit types. Our results imply that the biodiversity preservation and promotion of apricot landraces should not only rely on *ex-situ* strategies. It is worth developing a good balance between the commercial exploitation of specific fruit types and brands (important to strengthen socio-economic structures) and more inclusive biodiversity-based sustainable agriculture that can provide ecosystem services through an increased attractiveness of varietal mixtures [64].

## Figures and Tables

**Table 1:** Frequency (*f*) and relative frequency (rf) of the categorically scored traits in the germplasm collection. For each trait, phenotypes (Phen.) are ranked in decreasing order. For each qualitatively scored trait, the Simpson Index of Diversity (SDI) is also reported.

Tree Vigour (SDI: 0.52)				Fruit: Ground Colour of Skin (SDI: 0.48)				Fruit: Colour of Flesh (SDI: 0.70)				Kernel Bitterness (SDI: 0.07)				Fruit: Adherence of Stone to Flesh (SDI: 0.07)		
Phen. ^1^	*f*	rf		Phen. ^2^	*f*	rf		Phen. ^3^	*f*	rf		Phen. ^4^	*f*	rf		Phen. ^4^	*f*	rf
S	17	0.61		Y	18	0.64		LO	13	0.46		P	27	0.96		A-VW	27	0.96
M	3	0.11		LO	10	0.36		MO	7	0.25		A-VW	1	0.04		P	1	0.04
NA	3	0.11						C	6	0.21								
VS	3	0.11						DO	2	0.07								
W	2	0.07																

^1^ S: strong; M: medium; NA: not available/not consistent; VS: very strong: W: weak. ^2^ Y: yellowish; LO: light orange; R: red; O: orange; YG: yellowish-green; OR: orange-reddish; G: green. ^3^ LO: light orange; MO: medium orange; C: cream; DO: dark orange. ^4^ A-VW: absent-very weak; P: present.

**Table 2:** Selected descriptive statistics for the quantitative traits of the 28 apricot landraces. For each trait, the table reports the coefficient of variation (CV) and the maximum (max), average (mean), and minimum (min) value.

Trait (Abbreviation)	Unit	CV	Max	Average	Min
Fruit fresh weight (FFW)	g/fruit	18.4%	62.3	46.7	30.0
Fruit length (FL)	mm	9.9%	53.7	45.1	38.2
Fruit width (FW)	mm	8.9%	47.3	41.1	34.9
Fruit volume (FV)	cm^3^	26.3%	62.8	40.7	25.2
Solid Soluble Content (SSC)	°Brix	19.4%	22.7	15.9	10.4
Titratable acidity (TA)	g/L	38.0%	2.5	1.3	0.5
Flesh firmness (FF)	N	34.3%	54.0	29.0	17.7

**Table 3:** Main genetic indices of the apricot landraces obtained by SSR analysis. Na: number of different alleles; I: Shannon’s information index; Ho: observed heterozygosity; PIC: polymorphic information content; ENA: effective number of alleles; Ev: evenness F: Wright fixation index.

Locus	Na	I	Ho	PIC	ENA	Ev	F
AMPA095	4	1.06	0.75	0.61	2.55	0.82	−0.23
AMPA111	2	0.68	0.00	0.49	1.96	0.98	1.00
AMPA112	9	1.71	0.61	0.77	4.43	0.76	0.22
AMPA113	4	0.99	0.58	0.57	2.34	0.79	−0.01
AMPA124	7	1.32	0.48	0.64	2.79	0.65	0.25
UDAp-401	7	1.57	0.85	0.76	4.17	0.83	−0.11
UDAp-410	8	1.72	0.86	0.77	4.31	0.72	−0.12
UDAp-414	5	1.15	0.81	0.62	2.67	0.77	−0.30
UDAp-415	5	1.15	0.81	0.61	2.58	0.73	−0.33
UDAp-419	8	1.67	0.28	0.76	4.21	0.74	0.63
UDAp-420	7	1.49	0.68	0.72	3.52	0.73	0.05
UDAp-446	10	1.78	0.89	0.77	4.28	0.66	−0.16
Mean	6.33	1.36	0.63	0.67	3.32	0.77	0.07
Standard error	0.68	0.10	0.08	0.03	0.27	0.03	0.12

## Data Availability

Data are contained within the article or supplementary material. The raw data generated during and/or analysed during the current study are available from the corresponding author on reasonable request.

## Supplementary Materials

The following are available online at https://www.mdpi.com/article/10.3390/plants10071341/s1, Table S1: Phenotypic trait analysis. The full name of the variety is reported in the main article. Table S2: Matrix of the pairwise genetic distance between the landraces under investigation. See the main text for the landraces’ code. Table S3: SSR loci employed, primer sequences (in 5′ to 3′ order), and their main features.
