# Supplementary material for: Diversity and Relationships among Neglected Apricot (Prunus armeniaca L.) Landraces Using Morphological Traits and SSR Markers: Implications for Agro-Biodiversity Conservation
Source: Plants (Basel). 2021 Jun 30;10(7):1341. doi: 10.3390/plants10071341 (PMC8309161; doi:10.3390/plants10071341)
Supplement: Supplementary file 1 [file plants-10-01341-s001.zip › Table S1.pdf]

Supplementary Table S1: Phenotypic traits analysis. The full name of the variety is reported in the main body of the article.

Variety (code)	Fruit fresh weight (g/fruit)	Fruit length (mm)	Fruit width (mm)	Fruit volume (cm^3)	Solid Soluble Content (°Brix)	Titratable acidity (g/L)	Flesh firmness (N)	Tree: vigour	Fruit: ground colour of skin	Fruit: colour of flesh	Kernel: bitterness	Fruit: adherence of stone to flesh
ANT	54.5	53.7	45.8	34.34	15.5	1.20	34.34	strong	yellowish	light orange	present	absent or very weak
ARO	47.1	41.5	40.1	41.20	18.3	1.04	41.20	very strong	yellowish	cream	present	absent or very weak
BOC	40.5	43.7	38.1	52.68	16.1	1.44	52.68	strong	yellowish	dark orange	present	absent or very weak
CAF	53.0	44.6	44.2	38.26	15.9	1.34	38.26	strong	yellowish	cream	present	present
DIA	34.9	39.7	36.1	19.62	17.5	1.04	19.62	NA	light orange	light orange	present	absent or very weak
DON	62.3	44.9	44.8	24.53	13.1	2.48	24.53	strong	light orange	medium orange	present	absent or very weak
MAG	46.4	45.1	40.9	24.53	19.7	0.71	24.53	medium	yellowish	light orange	present	absent or very weak
MAM	37.9	38.2	38.4	34.04	16.57	1.41	34.04	strong	light orange	cream	present	absent or very weak
MON	61.5	49.8	45.9	19.62	16.62	0.85	19.62	strong	yellowish	light orange	present	absent or very weak
NON	44.1	42.9	39.0	17.66	16.88	0.95	17.66	strong	light orange	light orange	present	absent or very weak
PAN	45.3	45.3	40.5	27.47	18.5	1.12	27.47	strong	yellowish	medium orange	present	absent or very weak
PAO	51.0	50.8	42.8	44.15	17.5	1.43	44.15	weak	yellowish	light orange	present	absent or very weak
PAZ	50.6	47.0	44.8	53.96	10.9	1.05	53.96	very strong	yellowish	medium orange	present	absent or very weak
PEL	36.3	39.9	34.9	26.49	14.8	1.55	26.49	strong	yellowish	dark orange	present	absent or very weak
POR	49.9	44.5	43.9	20.60	20.6	0.45	20.60	NA	light orange	light orange	present	absent or very weak
PRE	46.4	45.5	39.4	18.64	12.6	NA	18.64	medium	light orange	cream	present	absent or very weak
RES	56.3	43.8	42.6	18.34	22.7	0.46	18.34	strong	light orange	light orange	present	absent or very weak
SAG	38.3	42.3	36.7	22.56	19.1	1.08	22.56	strong	yellowish	cream	present	absent or very weak
SAN	30.0	39.0	35.2	21.58	11.7	2.16	21.58	strong	yellowish	cream	present	absent or very weak
SCA	55.5	50.8	44.8	33.35	17.6	1.44	33.35	strong	yellowish	light orange	absent or weak	absent or very weak
SCH	47.7	53.7	47.3	36.30	10.4	1.21	36.30	strong	yellowish	medium orange	present	absent or very weak
SCI	43.9	44.7	41.1	20.60	18.7	0.64	20.60	NA	light orange	light orange	present	absent or very weak
SON	32.9	39.4	36.5	24.53	12.7	2.16	24.53	weak	light orange	light orange	present	absent or very weak
SOR	50.6	47.4	42.1	23.54	12.9	1.44	23.54	strong	yellowish	light orange	present	absent or very weak
STE	37.1	42.2	37.0	28.45	12.8	1.15	28.45	medium	light orange	light orange	present	absent or very weak

TAV	59.6	51.8	46.2	32.37	15.7	1.42	32.37	strong	yellowish	medium orange	present	absent or very weak
VIC	44.0	40.5	40.2	29.14	15.99	1.74	29.14	very strong	yellowish	medium orange	present	absent or very weak
ZEP	49.5	48.9	42.2	23.54	12.7	1.60	23.54	strong	yellowish	medium orange	present	absent or very weak
