# Supplementary material for: Diversity and Relationships among Neglected Apricot (Prunus armeniaca L.) Landraces Using Morphological Traits and SSR Markers: Implications for Agro-Biodiversity Conservation
Source: Plants (Basel). 2021 Jun 30;10(7):1341. doi: 10.3390/plants10071341 (PMC8309161; doi:10.3390/plants10071341)
Supplement: Supplementary file 1 [file plants-10-01341-s001.zip › Table S3.pdf]

Supplementary Table S3: SSR loci employed, primer sequences (in 5’ to 3’ order), and their main features.

Locus	Forward	Reverse	Core motif	Ta (°C)	Allelic size range (bp)	Reference
AMPA095	GCCAAAGTTAGGAAGGAGAATG	TCTCATATTCAAACCCCAATGAC	(AC)	56	79-87	[28]
AMPA112	CAAAGTGTCATTGTTCTTGCTAG	AAGGCCAAAACTCCAGTAGAAG	(AG)	55	206-236	[28]
AMPA111	ACTGACAGCAGCAGAATGTGTC	TATCATCAGGGCTTTTTGTGTG	(CTT)	55	171-207	[28]
AMPA113	CAACTCTGGTTTTCCTGTCTCTC	CCTTCAGACTAGCTGAGCTTCC	(CT)	55	116-152	[28]
AMPA124	TCAACCTGTAATAACCTAGCTTGC	AGCCGATGACTTGATTTTCCTC	(GA)	55	152-174	[28]
UDAp-401	AAACCCTAGCCGCCATAACT	GCTAAAGGCCTTCCGATACC	(TC)	56	152-176	[29]
UDAp-410	TTGTTGACAAGAAGAAAACAAAGC	CAACGGGTTGGTTTCAGAAG	(AG)	56	168-184	[29]
UDAp-414	CAAGCACAAGCGAACAAAAT	GGTGGTTTCTTATCCGATGC	(AG)	56	130-164	[29]
UDAp-415	AACTGATGAGAAGGGGCTTG	ACTCCCGACATTTGTGCTTC	(GA)	56	297-300	[29]
UDAP-419	TTCTTTTGGGATTGGTCTCG	GATTTTTAAATAACCAACCAGCTTC	(GA)	56	135-149	[29]
UDAp-420	TTCCTTGCTTCCCTTCATTG	CCCAGAACTTGATTCTGACCA	(CT)	56	96-140	[29]
UDAp-446	CCTCCCCCTAGATTTTCAGC	CGTGCTTGGGACATAGATCA	(GA)	54	150-180	[19]

Ta: temperature of annealing
